# Unsupervised Depth Completion Guided by Visual Inertial System and Confidence

**DOI:** 10.3390/s23073430

**Published:** 2023-03-24

**Authors:** Hanxuan Zhang, Ju Huo

**Affiliations:** School of Electrical Engineering and Automation, Harbin Institute of Technology, Harbin 150001, China

**Keywords:** confidence, unsupervised depth completion, loss function, dynamic scenes

## Abstract

This paper solves the problem of depth completion learning from sparse depth maps and RGB images. Specifically, a real-time unsupervised depth completion method in dynamic scenes guided by visual inertial system and confidence is described. The problems such as occlusion (dynamic scenes), limited computational resources and unlabeled training samples can be better solved in our method. The core of our method is a new compact network, which uses images, pose and confidence guidance to perform depth completion. Since visual-inertial information is considered as the only source of supervision, the loss function of confidence guidance is creatively designed. Especially for the problem of pixel mismatch caused by object motion and occlusion in dynamic scenes, we divide the images into static, dynamic and occluded regions, and design loss functions to match each region. Our experimental results in dynamic datasets and real dynamic scenes show that this regularization alone is sufficient to train depth completion models. Our depth completion network exceeds the accuracy achieved in prior work for unsupervised depth completion, and only requires a small number of parameters.

## 1. Introduction

Spatial awareness is an essential ability of robots, which can help robots perceive and understand various information in an unknown environment. The visual inertial system provides the robots with accurate self-positioning information and sparse environment maps through SLAM (simultaneous localization and mapping). However, these sparse depth maps obtained by SLAM cannot meet the complex task requirements of robots, such as path planning and collision avoidance [1].

Therefore, these sparse depth maps need to be converted into dense depth maps. Although there are some SLAM methods that can obtain dense depth maps in the environment, these methods are limited by various aspects. In recent years, depth completion methods have become popular in the field of robots. They can use the convolutional neural network method to complete the sparse depth map and RGB map into a dense depth map [2].

Depth completion methods are mainly divided into two types. One is the supervised methods that rely on the depth value for training [3,4,5,6], and the other is the unsupervised methods that are based on the principle of structure from motion (SFM) [7,8,9]. The supervised depth completion methods minimize the difference between the true depth and the predicted depth during training. Although these methods can guarantee the accuracy of training to the greatest extent, it needs to mark training samples pixel by pixel and frame by frame. This work requires a lot of manpower, and it is often very difficult to obtain the true depth. The unsupervised depth completion method avoids the complex work of marking training samples, but it is always challenged by SFM, such as dynamic objects and occlusion. In order to overcome these challenges, most scholars add additional supervision information such as optical flow and semantics, which make network parameters more complex [10,11]. In this paper, only monocular sequences and IMU (inertial measurement unit) data are used for supervision to realize deep completion and dynamic object motion joint unsupervised learning.

In summary, this work makes the following contributions:

(1) We design a tight coupling network of depth completion and motion residuals which can meet the real-time dense depth prediction of robots in dynamic complex environments with less computing resources.
(2) In the training process of our network, we do not need any auxiliary information except RGB images, pose information, and sparse depth maps provided by the visual-inertial system. That is, there is no need for semantic information, optical flow information, or any kind of ground truth.
(3) We propose a method to classify the image into static, dynamic, and occluded regions and design the loss function of each region separately. This method can solve the problem of dynamic object motion and occlusion in our network training, and can improve the precision of network output dense depth.
(4) In the KITTI depth completion dataset, the VIDO dataset, and the generalization in real dynamic scenes, our proposed method exceeds the accuracy achieved by the previous unsupervised depth completion work and requires only a small number of parameters. In addition, we also explicitly show the influence of confidence on the loss function, which can improve the data error until convergence.

## 2. Related Work

### 2.1. Supervised Depth Completion

The supervised depth completion methods mainly minimize the depth difference between the real pixel depth of each image and the depth predicted from RGB images and sparse depth in training [12]. The methods mainly focus on network structure modeling, loss function optimization, and parameter lightweight. Jaritz et al. presented a method to handle sparse depth data with optional dense RGB, and accomplished depth completion and semantic segmentation by changing only the last layer [13]. A CNN confidence propagation method to guide sparse depth regression was proposed in [14]. By introducing a normalized convolution layer and generating point-by-point continuous confidence maps to guide depth completion, the method achieves good accuracy. Based on [14], Teixeira et al. added a new confidence guidance network while maintaining the lightweight of network parameters and developed it in the embedded system [15]. Ref. [16] proposed the SemAttNet network, which guides depth completion through attention-based semantic perception. The features among the three branches of color orientation, semantic orientation, and depth orientation are fused based on SAMMFAB in this network. Ref. [17] proposed a lightweight depth completion network for depth perception in real-world environments. To effectively transfer a teacher’s knowledge that is useful for the depth completion, we introduced local similarity-preserving knowledge distillation (LSPKD), which allows for similarities between local neighbors to be transferred during the distillation. All these methods need to mark the true value of the training sample pixel by pixel, which is a very difficult and complicated task. We skipped these supervised methods, meaning to train the network through unlabeled samples to achieve the precision of supervised depth completion.

### 2.2. Unsupervised Depth Completion

Based on the principle of SFM, the unsupervised depth completion methods predict the depth information of pixels in the images by minimizing the photometric error of the input images and the reconstructed images. PnP (Perspective-n-Point) and RANSAC (random sample consensus) are used to align the luminosity term of the images and the second-order smoothness prior in [18]. However, Ma et al.’s approach can only be used for the depth completion of static scenes. Yang et al. used the nearest pixel correlation methods to learn conditional priors instead of using the local smoothing hypothesis [19]. In order to learn condition priori, a priori network needs to be introduced, so that the whole network is not a completely unsupervised network. Wong et al. used a predictive cross-modal criterion, akin to “self-supervision,” measuring photometric consistency across time, forward–backward pose consistency, and geometric compatibility with the sparse point cloud. On this basis, they imposed strong inductive biases on Euclidean reconstruction in the architecture and combined the calibrated back projection layer, so as to reduce the model size while maintaining the existing accuracy [20]. Similarly, these methods do not consider the interference caused by motion and occlusion of dynamic objects in the environment. In unsupervised depth completion methods, in order to overcome this adverse effect, the motion detection model [21] is usually added, as shown in Section 2.3.

### 2.3. Depth and Motion

When the cameras and dynamic objects move relative to the scene, to achieve consistency between views, it is necessary to estimate the motion state of the cameras and each object. Recently, several methods of learning depth, cameras motion, and object motion simultaneously from monocular sequences have been proposed. In [22], monocular depth estimation, camera self-motion estimation and continuous frame optical flow estimation are integrated into a network for unsupervised learning. Lu et al. designed a multitask learning framework, which can learn depth estimation and image segmentation unsupervised at the same time [23]. Depth and motion networks are designed in [24]. Li et al. proposed the motion regularization loss functions under the assumption that the dynamic objects are rigid bodies and only carry out translation motion. Although the above methods overcome the interference of dynamic objects, the network architectures become very complex. Different from the previous work, our method not only does not need to segment objects to estimate their motion states, but also designs regularization loss functions to overcome the disadvantage of mutual occlusion between dynamic objects and static scenes, which achieves the most advanced performance of unsupervised depth completion while greatly reducing the network parameters.

## 3. Methodology

**Figure 1:**
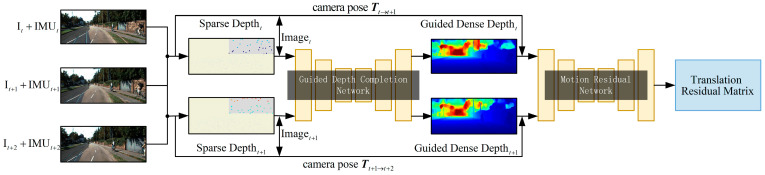
Overall training setup.

In this paper, we propose a method to accurately predict dense depth and object motion using only cameras and IMU sensors. Figure 1 describes the overview of our system, mainly including the guidance depth completion network and the motion residual network.

Specifically, we used VI-SLAM (see Section 3.1) to calculate the depth of feature points in the scene and the motion pose of the cameras. The sparse depth map and RGB images were used as the guidance depth complement network input to make the network output guidance dense depth (see Section 3.2). In addition, the dense depth, RGB images and camera motion pose were used as the input of the motion residual network to make the network predict the motion of each pixel relative to the background (see Section 3.2). We proposed a new regularized loss function (see Section 3.3), which is helpful for training in highly complex dynamic scenes.

### 3.1. VI-SLAM: Sparse Depth Map Generation

In this work, we used the method [25] to estimate camera pose and 3D feature point depth. Specifically, at each time frame, [25] extracted ORB features in the images, and tracked them by feature-matching through descriptors. Then, [25] fused IMU measurements for factor graph optimization to estimate camera motion and feature point depth. Each time an image was processed, [25] projected all visible 3D features onto the image, and used their depth to create a sparse depth on the metric scale. It was particularly emphasized that IMU plays an important role in the completion of metric depth. Without IMU, the VI-SLAM system cannot generate sparse depth maps on the metric scale.

### 3.2. Depth and Motion Networks

The depth and Motion networks are mainly divided into two parts. The first part is the guidance depth completion network used to estimate the dense depth, and the second part is the motion residual network used to estimate the object motion. 

**Figure 2:**
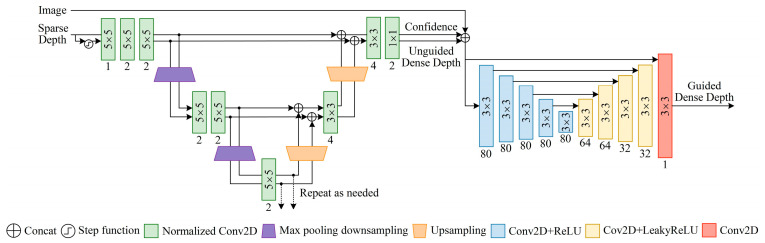
Depth completion network. The sparse depth maps and RGB images are used as inputs, and the input confidence level in the normalized convolution is objected by calculating the binary mask with the step function. Finally, the dense depth maps guided by confidence are the output.

Inspired by Eldesokey et al. [14], Figure 2 shows a new network of depth completion. This is a layered multi-scale architecture. The architecture acts as a generic estimator for different scales and gives a good approximation for the dense output at a very low computation cost. In the first stage, we used the normalized convolution model. The concept of normalized convolution was proposed by Knutsson and Westin based on the signal theory with confidence [26]. The core advantage of normalized convolution is the separation between signal (depth) and confidence. We only needed to provide an initial confidence. Through continuous normalization convolution, we can adaptively process this confidence and determine the final confidence of the output and the unguided dense depth. At the same time, the output confidence can also reflect the density of input confidence. We refer to the method of outputting confidence in the literature [27]. In our network, in order to obtain the initial confidence, we calculated the step function of the sparse depth image. That is, the confidence level for pixels with depth value was set to 1, and the confidence level for pixels without depth value was set to 0. In addition, in order to fuse different scales, the output and corresponding confidence of the last normalized convolution layer were unsampled using the nearest neighbor interpolation, and connected with the corresponding scale through jump connection. Because of the dense depth learned from the sparse depth images estimated by VI-SLAM, it shows weakness in the local area of weak textures. Therefore, this problem was mitigated in the second stage by using RGB images and new forms of auxiliary data fusion. The auxiliary data were the confidence level of the first stage output that holds the reliability of the depth value of each pixel. We will prove in subsequent experiments that the accuracy of depth complement is improved by assisting RGB image fusion with output confidence.

The input of the motion residual network is a pair of consecutive frames connected along the channel dimension. The motion residual network is similar to the network in reference [28]. The difference is that each input image has five channels: RGB channel, guided dense depth channel and motion pose channel. The output of the motion residual network is the translation residual matrix $t^{obj}(\delta u,\delta v)$, which represents the translation size of the dynamic objects in the pixel coordinate system.

According to the dense depth ${\hat{D}}_{t}$ of the current frame outputted by the guidance depth complement network, and the motion pose of two adjacent frames provided by visual-inertial system, the pixel coordinate ${\hat{P}}_{t+1}$ of each pixel point of the current frame in the next frame image are:

(1) $${\hat{z}}_{t+1}{\hat{P}}_{t+1}=KT_{t}^{t+1}{\hat{D}}_{t}K^{-1}P_{t},{\hat{P}}_{t+1}=({\hat{u}}_{t+1},{\hat{v}}_{t+1})$$

where K is the internal parameter of the cameras, and ${\hat{z}}_{t+1}$ is the depth value of each pixel of the predicted next frame image.

Equation (1) only considers the pixel change caused by camera motion, and does not consider the pixel change between two frames caused by the motion of the dynamic object itself in the scene. As shown in Equation (2), the accurate pixel coordinate ${\hat{P}}_{t+1}^{obj}$ and the corresponding pixel value $I({\hat{P}}_{t+1}^{obj})$ of the next frame image of the dynamic object can be obtained through the translation residual matrix output by the motion residual network.

(2) $${\hat{P}}_{t+1}^{obj}=({\hat{u}}_{t+1}+\delta u_{i},{\hat{v}}_{t+1}+\delta v_{i})$$

**Figure 3:**
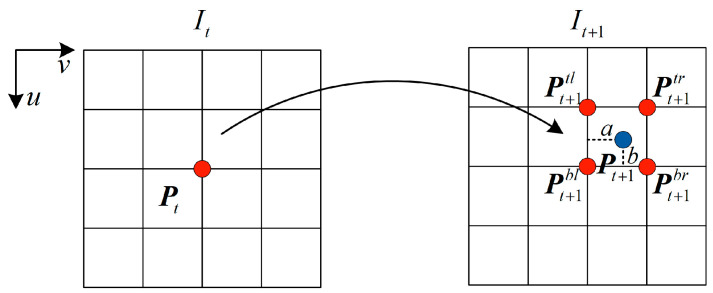
Diagram of bilinear interpolation process.

Meanwhile, as shown in Figure 3, in the process of projection reconstruction between two frames, the pixel value I(P) of each pixel point of the reconstructed images can be obtained through the differentiable bilinear interpolation method [25]:

(3) $$I\left(P\right)=(1-a)(1-b)I\left(P_{}^{bl}\right)+a(1-b)I\left(P_{}^{br}\right)+abI\left(P_{}^{tl}\right)+(1-a)bI\left(P_{}^{tr}\right)$$

### 3.3. Losses

Since our goal was to only use VIO for depth completion and dynamic object motion learning in dynamic scenes, a lot of regularization of loss functions was required during training. In particular, we explicitly considered the problem of occlusion in order to eliminate the occlusion interference in the unsupervised training of depth completion, and designed the corresponding loss functions for different situations in the occlusion area. Starting from analyzing different situations of occluded areas, we describe the following loss functions, which are the key contributions of this work.

**Figure 4:**
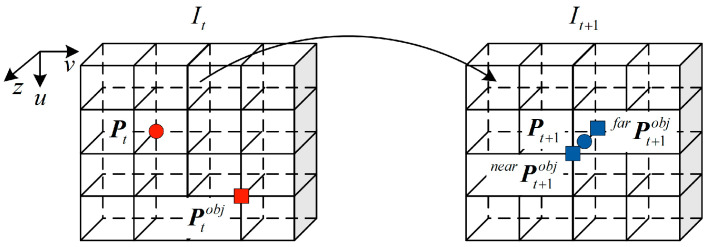
Diagram of mutual occlusion between dynamic objects and static structures. When a dynamic object and a static structure or multiple dynamic objects are in the same pixel, the pixel belongs to the one with the lowest depth.

Through the RGB images and the translation residual output of motion residual network, the pixel coordinate $P_{t}^{obj}$ and the corresponding pixel value $I\left(P_{t}^{obj}\right)$ of the dynamic object at the current time can be obtained. The dynamic object area at the current time is defined as a dynamic area, and the static structure area at the current time is defined as a static area. When the current frame images are projected to the next frame, due to the motion of the cameras and the dynamic objects, there will be an area occluded by each other. This area is defined as an occluded area. As shown in Figure 4, occlusion conditions in the occlusion area are mainly divided into two types: the depth of static structures are smaller than dynamic objects and the depth of static structures are larger than dynamic objects.

According to Equation (2), the pixel coordinate ${\hat{P}}_{t+1}^{obj}$ of the next frame image of the dynamic objects and the pixel value $I({\hat{P}}_{t+1}^{obj})$ of the corresponding RGB images can be obtained. Note that the key point at this time is to determine whether the pixel belongs to the dynamic object or the static scene. According to Equation (4), the pixel coordinate ${\hat{P}}_{t+1}^{obj}$ can be projected back to the pixel coordinate ${\hat{P}}_{t}$ of the current frame, and the corresponding pixel value $I({\hat{P}}_{t})$ can be obtained from the RGB images.

(4) $${\hat{z}}_{t}{\hat{P}}_{t}=KT_{t+1}^{t}{\hat{D}}_{t+1}K^{-1}{\hat{P}}_{t+1}^{obj}$$

Next, we identified the category of occlusion by judging the pixel difference between $\left|I({\hat{P}}_{t+1}^{obj})-I({\hat{P}}_{t})\right|$ or $\left|I({\hat{P}}_{t+1}^{obj})-I(P_{t}^{obj})\right|$. If the former is smaller than the latter, then the static structure depth is small. That is, static structures are not occluded and dynamic objects are occluded. At this point, pixels in the occluded area are naturalized to pixels in the static area. On the contrary, the static structure depth is larger, and the pixels of the occluded area are naturalized to pixels of the dynamic area. Similarly, when multiple dynamic objects move to the same pixel, the pixel difference of these dynamic objects and static structures can be used to identify which of them pixel value of the pixel belongs to.

**Figure 5:**
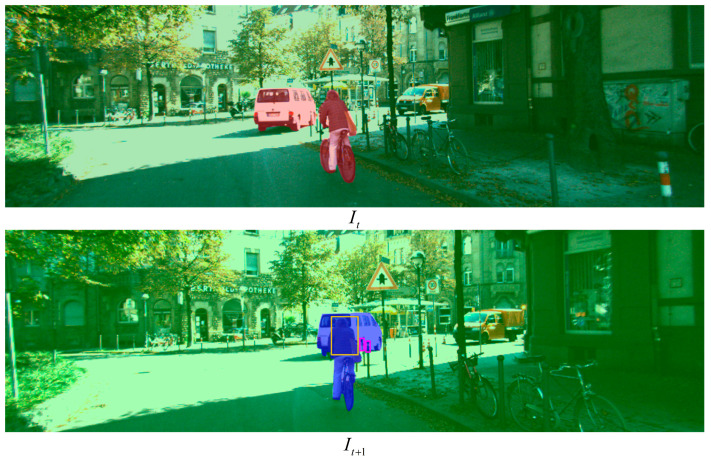
Schematic diagrams of pixel region classification of two adjacent frames. The red shadow represents the dynamic region at the current time, and the green shadow represents the static region. The blue shadow represents the area to be occluded at the next moment. The pink box indicates that static structures occlude dynamic structures in the area, the orange box indicates that dynamic structures occlude each other, and the rest are dynamic structures occlude static structures.

As shown in Figure 5, we listed a group of schematic diagrams of pixel region classifications of two adjacent frames. Through the motion residual network, the dynamic area at the current time can be obtained (marked by red shadow), and the static area at the current time can be marked by green shadow. At the next moment, we can judge the occluded area (marked by blue shadow) through the above description, in which the pink box is static structure occluding dynamic structure, the orange box is dynamic structure occluding each other, and the rest is dynamic structure occluding static structure. In training, the pixels in the pink box belong to the static area, and the rest belong to the pixels in the dynamic area.

The translation residual $t^{obj}(\delta u,\delta v)$ of the residual translation network output is regularized normalized by the $L_{m}^{d}$ loss function. In order to keep the motion of all pixels belonging to a dynamic object consistent, the translation difference of each dynamic object in the dynamic region using the $L_{m}^{d}$ loss function is minimized. It is defined as:

(5) $$L_{m}^{d}=L_{1}(\sum \iint \sqrt{{\left(\partial_{u}t^{obj}(\delta u_{i},\delta v_{i})\right)}^{2}+{\left(\partial_{v}t^{obj}(\delta u_{i},\delta v_{i})\right)}^{2}}dudv)$$

The dense depth ${\hat{D}}_{t}$ of the depth complement network output is normalized by the photometric consistency losses and parallax smoothing losses that are complementary to the maximum confidence. The luminosity loss consistency is a combination of $L_{1}$ penalty and SSIM [29] for average reprojection error of each pixel, and SSIM is a perceptual measure of the constant change of local illumination. Parallax smoothing losses are the standard edge perception smoothing regularization of parallax map $d_{u,v}$. It ensures that the loss weight of parallax smoothing is small when the images gradient is large. Specifically, the photometric consistency losses are calculated as shown in Equation (6) for pixels in the static region. The photometric consistency losses are calculated as shown in Equation (7) for pixels in the dynamic region. The parallax smoothing losses are calculated as shown in Equation (8) for all pixel points.

(6) $$L_{vc}^{s}=\frac{1}{2N}\sum_{N}|I(P_{t})-I({\hat{P}}_{t+1})|+(1-\mathrm{SSIM}(I(P_{t}),I({\hat{P}}_{t+1})))$$

(7) $$L_{vc}^{d}=\frac{1}{2N}\sum_{N}\left|I({\hat{P}}_{t+1}^{obj})-I(P_{t}^{obj})\right|+(1-\mathrm{SSIM}(I({\hat{P}}_{t+1}^{obj}),I(P_{t}^{obj})))$$

(8) $$L_{ds}=\frac{1}{N}\sum_{u,v}(|\partial_{u}d_{u,v}|e^{-\left\|\partial_{u}I_{u,v}\right\|}+|\partial_{v}d_{u,v}|e^{-\left\|\partial_{v}I_{u,v}\right\|})$$

In the supervised depth completion network with normalized convolution layer, it is necessary to maximize the output confidence $C_{u,v}$ while minimizing the data error. Therefore, it is necessary to realize the loss functions of these two objectives at the same time. The total losses ${\tilde{L}}_{E}$:

(9) $$L_{E}=L_{vc}^{s}+L_{vc}^{d}+0.25\times L_{ds}$$

(10) $${\tilde{L}}_{E}=L_{E}-\frac{1}{p}(C_{u,v}-L_{E}C_{u,v})$$

where p is the epoch number. The confidence term is decaying by dividing it by the epoch number p to prevent it from dominating the losses when the data error term starts to converge.

## 4. Experiments and Discussion

In this section, in order to demonstrate the capability of our proposed method, we compared it with other advanced depth completion networks on the KITTI depth completion dataset [30] and VOID dataset [20]. Moreover, the weight model based on VOID dataset training is used in real scenes to verify the generalization ability of our method. We adopted the standard evaluation indicators of the KITTI depth completion dataset: mean absolute error (MAE) and root mean square error (RMSE), calculated according to the depth value. MAE is an unbiased error estimation, which estimates the average error of the whole image, while RMSE punishes the outliers. In addition, we also used iMAE and iRMSE, which are calculated based on parallax rather than depth. The four evaluation indicators are defined in Table 1.

### 4.1. Datasets and Setup

The KITTI depth completion dataset provides raw image frames and associated sparse depth maps of approximately 80,000 outdoor dynamic scenes. The sparse depth maps are made up of a point-cloud depth of about 5% density output by Velodyne lidar sensors. In order to better verify the performance of our algorithm, we used the sparse depth map of about 0.5% density generated by the VI-SLAM method. The sparse depth maps were obtained through the methods discussed in Section 3.1 and the raw data were provided by the KITTI dataset. We used 1000 officially selected test samples for verification and compared our method with other advanced supervised/unsupervised depth completion methods.

The VOID dataset includes synchronized 640 × 480 RGB images (laboratories, corridors, classrooms, and gardens) and sparse depth maps of about 0.5% density. It is worth emphasizing that the sparse depth points provided by the dataset are a set of features tracked by the VI-SLAM system, which fully match the sparse depth map required by our depth complement network. The whole dataset is divided into training samples of 48 sequences and test samples of 8 sequences (including 800 frame images and sparse depth maps). We still used the evaluation scheme of the KITTI dataset (see Table 1) in order to better compare our method with it. 

The OpenLORIS dataset [31] is obtained from daily life scenes, and it is the first large-scale dataset containing indoor dynamic scenes. The dataset contains 848*480 RGB images, aligned depth images, and IMU information collected by Inter-D435i. It is noteworthy that this dataset does not modify the collected images, and it can be used as a test set of real scenes. In order to verify the generalization ability of our method in real scenes, we used the model trained by the VOID dataset to test in different scenes (cafe, home, and market).

The proposed method was implemented using PyTorch and trained on a machine equipped with an NVIDIA GTX 3090 Ti GPU with 24GB memory. For all our experiments, we adapted the Adam optimizer, starting with a learning rate of 10^−4^ and reducing it by a factor of 10 every three epochs down to 10^−6^. We let the model train for 24 h and reported the best epoch. We used the standard PyTorch weights for ResNet and batch size 8. In view of the diversity of depth in the dataset, it was necessary to standardize the sparse depth before inputting it into the network. We used sparse depth maps of about 0.5% density, which were generated by VI-SLAM (KITTI dataset and OpenLORIS dataset), as shown in Section 3.1 and provided by VOID.

### 4.2. Comparison of the KITTI Depth Completion Dataset

We submitted the proposed method to the KITTI Depth benchmark and compared it with advanced unsupervised and supervised methods. Among them, the MDPC method proposed by Liu et al. is an adaptive knowledge distillation method, which integrates some existing excellent models while avoiding some errors in the models [32]. Among the supervised depth completion methods, the methods with higher accuracy are the MFF-Net method by Liu et al. [33] and the NLSPN method by Park et al. [12]. The MFF-Net method uses multimodal feature fusion to achieve monocular depth completion. The NLSPN method proposes a robust and efficient end-to-end nonlocal spatial propagation network for depth completion. By introducing a learnable affine normalization method, it provides better robustness to mixed depth problems with depth boundaries. The MFF-Net method performs better in terms of RMSE, representing fewer outliers in its overall depth-completion results. The NLSPN method performs better in terms of MAE, representing less mean error in its completion results. 

The quantitative results of the KITTI depth dataset test set, using the above evaluation indicators and methods, are shown in Table 2. The supervised depth completion methods perform better than the unsupervised depth completion methods overall. Our method is superior to the most advanced unsupervised depth completion methods and some supervised depth completion methods, and slightly worse than the most advanced supervised depth completion methods. In addition, compared with the most advanced unsupervised MDPC method [32] in Table 2, our method can improve the MAE by 1.93%, RMSE by 1.76%, iMAE by 3.26% and iRMSE by 3.79%. It is worth emphasizing that the sparse depth input to our network is generated by VI-SLAM, which only accounts for 0.5% of the pixel density of the images, while other methods use the sparse depth maps with the built-in density of 5% in the dataset. Better depth completion effects are obtained, although only sparse depth maps with less density are utilized in the proposed method. In addition, the number of parameters and the running time of the network are discussed in detail in Section 4.5.

Next, we discuss the influence of input sparse depth maps with different densities of sparsity on our depth completion results. Sparse depth maps of 0.05% and 0.15% are obtained through sparsity operations. Through the deep completion results of three sparsity degrees (Table 3), we can see that our method can still obtain reasonable results. As the densities of sparsity decrease, the process of network reconstruction and depth completion becomes more and more difficult, resulting in a decline in performance. However, our performance degrades gracefully, and when the density decreases by a factor of 10, our error only doubles. This is because our method also uses pose information in guiding deep completion, which can overcome the reconstruction difficulties to a large extent.

**Figure 6:**
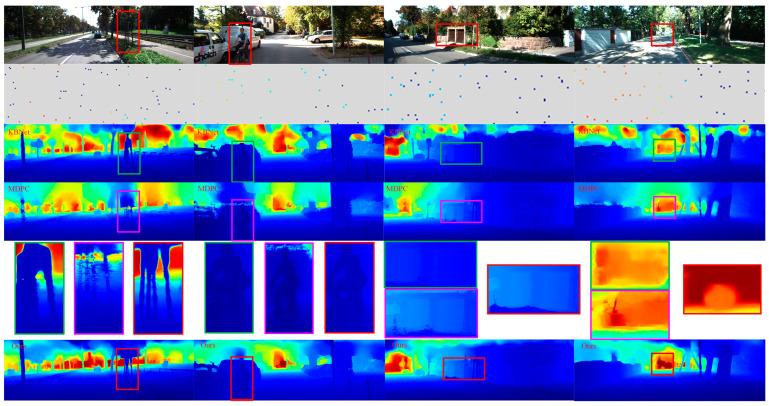
Qualitative results on the KITTI test set. Head-to-head comparison against [9,32]. Our method performs better in the regions with depth-mixing of small structures and the regions with obvious photometric changes in the distance. Note that the sparse depth images are enlarged for visualization and only the areas highlighted in red are shown. In line 5, the green boxes are partially enlarged images of the KBNet results, the pink boxes are partially enlarged images of the MDPC results, and the right red boxes are partially enlarged images of the results of our method.

To display the improvements from our contributions, we show a positive qualitative comparison with advanced unsupervised method [9,32] in Figure 6. The first line is RGB images, the second line is sparse depth maps generated by VI-SLAM (the size of depth is represented by different colors), the third line is KBNet method proposed by wong et al., the fourth line shows the MDPC method proposed by Liu et al., the fifth line shows the local magnification comparison map, and the sixth line shows our method, where challenging regions are highlighted. Our method performs better in the regions with depth-mixing of small structures and the regions with obvious photometric changes in the distance. This is due to the sparse depth generated by our VI-SLAM and the guidance of the confidence network. The former can better extract the depth information of tiny details and bright landmarks, while the latter can better guide 2D images of topology to 3D position coding.

### 4.3. Comparison of the VOID Dataset

In Section 4.2, our method tests well the effect of outdoor depth completion on the KITTI depth completion dataset. Because there are many complex scene layouts and weakly textured areas in indoor scenes, it was very important and meaningful to test our depth completion method in the VOID dataset based on indoor scenes.

According to the evaluation indexes in Table 1, our method is compared with five excellent unsupervised depth completion methods and the most advanced supervised depth completion methods. The comparison results are shown in Table 4. Our method achieves the best results in unsupervised methods. Specifically, it is superior to the MDPC method [29], with 17.05% MAE, 4.88% RMSE, 26.65% iMAE, and 16.92% iRMSE. Compared with the most advanced supervised depth completion method, our method is 5.52% more RMSE. Meanwhile, our method is much better than this supervised method in terms of completion time. For details, please refer to Section 4.5.

In this section, we continue to explore the impact of inputting depth maps with different sparsity densities on our depth completion network. As shown in Table 5, our accuracy is still guaranteed at a sparse depth map of 0.05%. This further proves that our method will become a common depth completion method under harsh conditions without providing additional information.

**Figure 7:**
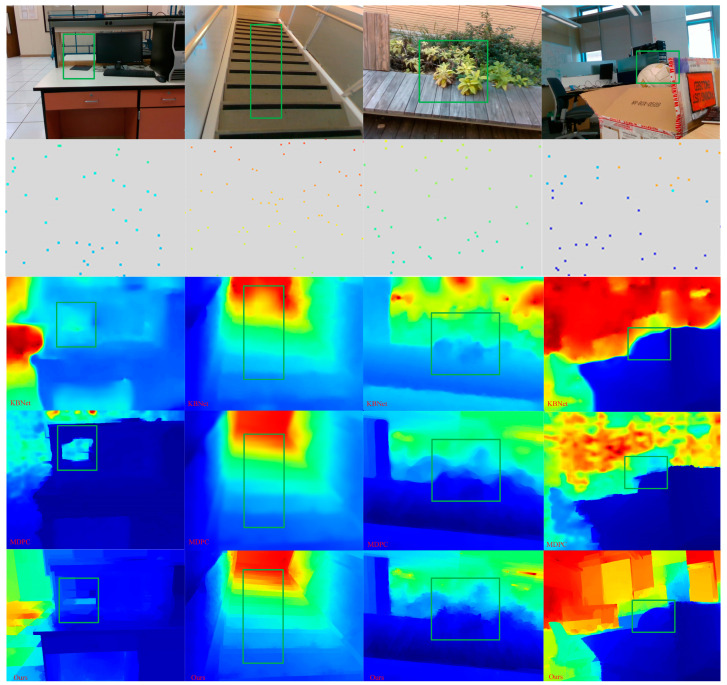
Qualitative results on the KITTI test set. Head-to-head comparison against [9,32]. Our method is smoother and more consistent in terms of edge sharpness and level of detail. Note that the sparse depth images are enlarged for visualization and only the areas highlighted in green are shown.

As shown in Section 4.2, we have selected challenging scenarios in the VOID dataset and conducted some qualitative examples. As shown in Figure 7, our method, both the KBNet method and the MDPC method, can conduct a very good reconstruction of a dense map from sparse to dense. However, in terms of edge sharpness and level of detail, our prediction and reconstruction are smoother and more consistent, especially along the edge. This is due to the fact that we proposed a confidence propagating method and fusion strategy between CNN layers, so that continuous confidence values of depth points can be generated for the output of the depth network and the edge structure information can be reconstructed to the maximum extent by combining the depth and RGB information. This is the highlight of our approach.

### 4.4. The Impact of the Proposed Losses

**Figure 8:**
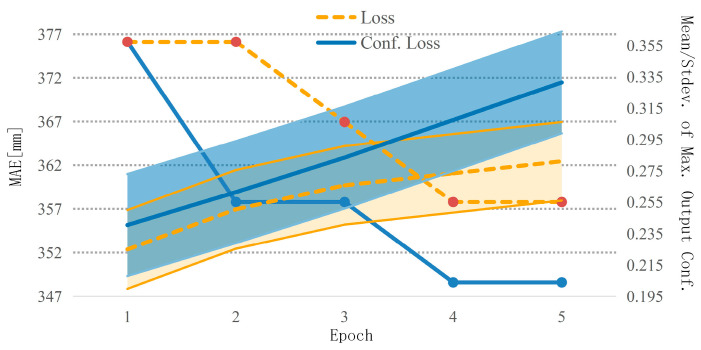
The impact of the proposed loss on confidence levels. Orange represents the loss function without confidence guidance, and blue represents the loss function with confidence guidance. The right axis represents the mean and standard deviation of maximum output confidence value over all images, while the left axis has the MAE in millimeters. When using a loss with only a data term, output confidence levels are lower, while our proposed loss achieves monotonically increasing confidence levels as well as a lower MAE. Note that the shaded area represents the standard deviation.

In order to study the role of confidence terms in guiding the training of the depth completion network, that is, the influence of confidence terms on the proposed loss function (Equation (10)), we trained our network twice. The loss function with confidence terms was used the first time (Equation (10)), and the loss function without confidence terms was used the second time (Equation (9)). Figure 8 shows the mean and standard deviation of the maximum output confidence of the images in the KITTI dataset on the right axis and the MAE error on the left axis. A monotonically increasing confidence map was generated through our loss-training network, while improving the depth completion error until convergence. On the other hand, if the network was not trained with confidence guidance, it had a lower output confidence level and converged to a higher MAE.

Next, as shown in Table 6, we conducted ablation research on each item in the loss function to verify the role of each part in training. 

As shown in Table 6, the loss functions designed for both dynamic and static regions can well help the network to complete the dense depth. Better completion accuracy is obtained when the loss functions of two regions are used simultaneously. At the same time, adding parallax smoothing loss and confidence term guidance in training can also effectively reduce the depth completion error. In addition, we also tested the weight of parallax smoothing loss in the whole loss function. We can find that the overall training effect is more satisfactory when the weight is 0.25.

### 4.5. The Number of Parameters and Runtime Comparison

The number of parameters and running times of some advanced methods on the KITTI depth complete dataset are compared in this section. They are taken from the relevant papers and the KITTI benchmark server.

Table 7 shows that our number of parameters ranks third. Compared with the first two networks developed for embedded systems with limited computing resources, our number of parameters is relatively small, which can also be applied to embedded systems of robots. In addition, it only takes 0.03 s for our method to complete a dense depth map from a sparse depth map with a density of about 0.5%. Among all the methods, our method also has the advantage of being a method with high potential for maintaining real-time performance. The supervised NLSPN method, which achieves the best test results in 4.2 and 4.3, takes 0.2 s to complete a sparse depth image. This is a depth completion method that cannot be developed on a real-time system. Please note that the quantitative results in Table 2 and Table 4 show the efficiency of our proposed method. This is because we apply the normalized convolution layer and then perform the downsampling. At the same time, we integrate different scales and use the nearest neighbor interpolation to perform the upsampling. This process makes full use of the characteristics of each scale to the maximum extent, so that the prediction accuracy of the network can still be maintained when the network parameters are reduced.

### 4.6. Generalization Capability on Real Scenes

In order to further verify the generalization ability of our method in real scenes, we, respectively, applied the model of our method and the literature [9,32] trained in VOID dataset to the depth completion of OpenLORIS dataset and compared them. We selected 1000 images from three complex real scenes of cafe, home, and market as the test, and used the provided IMU data and RGB images to generate sparse depth images with a density of 0.5%, ensuring the same input sources for the three methods.

**Figure 9:**
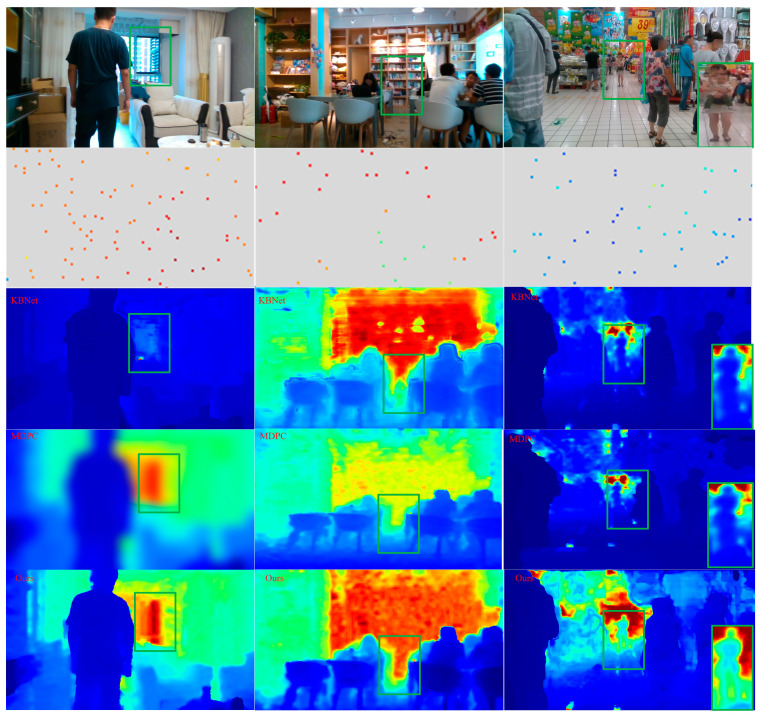
Qualitative results on generalization in real scenes. Head-to-head comparison against [9,32]. Our method is better in generalization ability. Note that the sparse depth images are enlarged for visualization and only the areas highlighted in green are shown.

The qualitative results of the three methods are shown in Figure 9. It can be clearly seen from the marked places that our method is more accurate in depth estimation, and better in edge sharpness and details (building outside the window, children in arms of adults) after depth completion, which proves that our generalization ability is stronger. The other two methods are relatively weak in generalization capability.

In order to quantitatively obtain the error of our method generalization in the real scenes, we took the depth images provided by the depth cameras as the true value, evaluated the completion accuracy of our method, and compared it with the literature [9,32]. The comparison results are shown in Table 8. Consistent with our qualitative results, our method obtains good completion accuracy and is superior to the KBNet method [9] and MDPC [32].

## 5. Conclusions

We propose a new unsupervised depth completion method for depth completion in highly dynamic scenes, which jointly solves the translation residual matrixes and dense depth maps. In order to solve the adverse effects caused by the motion and occlusion of dynamic objects in training, we divided the images into static, dynamic, and occluded regions, and designed loss functions guided by the visual inertial system and confidence, respectively. The loss function can simultaneously minimize the data error and maximize the output confidence. Finally, by combining the depth with confidence and RGB information to fuse the structural information, the dense depth maps after completion were more accurate in edge sharpness and detail. We comprehensively evaluated our method on the KITTI depth completion dataset, and the VOID dataset and generalization ability of real scenes. Compared with the most advanced unsupervised depth completion method, we achieved excellent performance with fewer network parameters.

## Figures and Tables

**Table 1:** Error metrics.

Metric	Units	Definition
MAE	mm	$\frac{1}{N}\sum \left|D_{pred}-D_{gt}\right|$
RMSE	mm	${\left(\frac{1}{N}\sum {\left|D_{pred}-D_{gt}\right|}^{2}\right)}^{1/2}$
iMAE	1/km	$\frac{1}{N}\sum \left|\frac{1}{D_{pred}}-\frac{1}{D_{gt}}\right|$
iRMSE	1/km	${\left(\frac{1}{N}\sum {\left|\frac{1}{D_{pred}}-\frac{1}{D_{gt}}\right|}^{2}\right)}^{1/2}$

**Table 2:** Quantitative results of the KITTI test dataset.

Method	Supervised	MAE	RMSE	iMAE	iRMSE
Ma [18]	No	350.32	2312.57	2.05	7.38
Yang [19]	No	343.46	1299.85	1.57	4.07
Wong [20]	No	299.41	1169.97	1.20	3.56
Wong [9]	No	256.76	1069.47	1.02	2.95
Liu [32]	No	218.60	785.06	0.92	2.11
Eldesokey [14]	Yes	233.26	829.98	1.03	2.60
Teixeira [15]	Yes	218.57	737.49	0.97	2.31
Park [12]	Yes	199.59	741.68	0.84	1.99
Liu [33]	Yes	210.55	719.98	0.94	2.21
Ours	No	**214.38**	**771.23**	**0.89**	**2.03**

**Table 3:** Depth completion of the KITTI test dataset with varying sparse depth densities.

Method	Density	MAE	RMSE	iMAE	iRMSE
Ours	0.5%	**214.38**	**771.23**	**0.89**	**2.03**
	0.15%	362.91	1358.14	1.65	3.87
	0.05%	420.73	1536.49	1.90	4.35

**Table 4:** Quantitative results of the VOID test dataset.

Method	Supervised	MAE	RMSE	iMAE	iRMSE
Ma [18]	No	198.76	260.67	88.07	114.96
Yang [19]	No	151.86	222.36	74.59	112.36
Wong [20]	No	73.14	146.40	42.55	93.16
Wong [9]	No	39.80	95.86	21.16	49.72
Liu [32]	No	36.42	87.78	19.18	43.83
Park [12]	Yes	26.74	79.12	12.70	33.88
Ours	No	**30.51**	**83.49**	**14.27**	**36.41**

**Table 5:** Depth completion on VOID with varying sparse depth densities.

Method	Density	MAE	RMSE	iMAE	iRMSE
Ours	0.5%	30.15	83.49	14.27	36.41
	0.15%	65.41	153.90	40.75	94.17
	0.05%	76.92	174.26	51.84	105.03

**Table 6:** The impact of the proposed losses in the KITTI depth completion dataset.

Methods	Losses	Confidence	MAE
Ours	$L_{vc}^{s}$	No	305.74
	$L_{vc}^{d}$	No	330.56
	$L_{E}=L_{vc}^{s}+L_{vc}^{d}$	No	238.12
		Yes	227.99
	$L_{E}=L_{vc}^{s}+L_{vc}^{d}+0.15\times L_{ds}$	No	231.05
		Yes	223.47
	$L_{E}=L_{vc}^{s}+L_{vc}^{d}+0.20\times L_{ds}$	No	226.34
		Yes	218.02
	$L_{E}=L_{vc}^{s}+L_{vc}^{d}+0.25\times L_{ds}$	No	226.51
		**Yes**	**214.38**
	$L_{E}=L_{vc}^{s}+L_{vc}^{d}+0.30\times L_{ds}$	No	224.78
		Yes	217.91
	$L_{E}=L_{vc}^{s}+L_{vc}^{d}+0.35\times L_{ds}$	No	230.43
		Yes	222.65

**Table 7:** Number of parameters and runtime for some methods in comparison (lower is better).

Method	#Params [M]	Runtime [s]
Ma [18]	27.8	0.08
Wong [20]	9.7	0.04
Wong [9]	6.9	0.02
Liu [32]	2.3	0.02
Eldesokey [14]	0.356	0.02
Teixerira [15]	0.5	0.06
Park [12]	25.84	0.2
Ours	**1.17**	**0.03**

**Table 8:** Quantitative results of generalization capability on real scenes.

Method	Supervised	MAE	RMSE	iMAE	iRMSE
Wong [9]	No	90.13	164.52	24.08	41.26
Liu [32]	No	77.69	140.17	18.55	35.04
Ours	No	**42.85**	**94.73**	**11.31**	**20.41**

## Data Availability

The data that support the findings of this study are available from the corresponding author, J.H., upon reasonable request.
